# Targeting NOP14 remodels the tumor immune microenvironment and enhances the antitumor efficacy of PD-1 blockade in DLBCL

**DOI:** 10.3389/fimmu.2026.1799719

**Published:** 2026-07-22

**Authors:** Lu Zou, Yanxiao Xiang, Junjun Wang, Chuanpeng Liang

**Affiliations:** 1 Department of Pharmacy, Qilu Hospital of Shandong University, Jinan, Shandong, China; 2 School of Basic Medical Sciences, Cheeloo College of Medicine, Shandong University, Jinan, Shandong, China; 3 High Magnetic Field Laboratory, Key Laboratory of High Magnetic Field and Ion Beam Physical Biology, Hefei Institutes of Physical Science, Chinese Academy of Sciences, Hefei, Anhui, China

**Keywords:** DLBCL, lipid nanoparticles, NOP14, prognosis, tumor immune microenvironment

## Abstract

**Background:**

Nucleolar Protein 14 (NOP14), a highly conserved factor in eukaryotes, is essential for pre-18S rRNA processing and small ribosomal subunit assembly. Emerging evidence underscores its pivotal regulatory role in the progression of various malignancies. Nevertheless, its functional involvement in Diffuse large B-cell lymphoma (DLBCL), particularly its capacity to modulate the tumor immune microenvironment, remains to be fully elucidated.

**Methods:**

Genomic and clinical data from DLBCL patients were retrieved from the GEO database (GSE12195, GSE32018, GSE181063 and GSE10846) for integrated bioinformatics analysis. NOP14 expression was quantified via qRT−PCR, western blot, and immunohistochemistry (IHC). Stable NOP14-knockdown cell lines were established using lentiviral vectors to assess proliferation through EdU, colony formation, and CCK−8 assays *in vitro*, as well as in the A20 syngeneic mouse model of DLBCL *in vivo*. To explore the underlying mechanism of NOP14, RNA sequencing was performed followed by KEGG enrichment analysis. Furthermore, flow cytometry was utilized to determine the correlation between NOP14 expression and the infiltration of immune cells. Finally, we developed cRGD-modified lipid nanoparticles encapsulating NOP14-siRNA (NOP14-siRNA@LNP) to assess its synergistic potential with anti-PD-1 therapy.

**Results:**

NOP14 was significantly overexpressed in DLBCL tissues and cell lines, with elevated levels correlating with shorter overall survival and unfavorable clinicopathological features. Our findings reveal that NOP14 exerts a prominent dual role in driving tumor proliferation and facilitating immune evasion in DLBCL. Characterized as a cell-intrinsic oncogene, NOP14 directly sustains tumor growth, whereas its depletion significantly compromises DLBCL cell proliferation both *in vitro* and *in vivo*. Concurrently, NOP14 functions as an extrinsic regulator of the tumor microenvironment by impairing CD8^+^ T cell infiltration and cytotoxic function to mediate immune escape. Mechanistically, NOP14 ablation inactivates the Wnt/β-catenin/c-Myc signaling cascade, which subsequently downregulates PD-L1 expression. This molecular shift successfully reverses the immunosuppressive state, markedly boosting tumor-infiltrating CD8^+^ T cells and stimulating their IFN-γ production. Notably, the specific depletion of CD8^+^ T cells significantly attenuated the effect of NOP14 on tumor growth. Additionally, cRGD-modified NOP14-siRNA@LNP successfully targets DLBCL via αvβ3 integrin recognition with excellent biocompatibility. NOP14-siRNA@LNP monotherapy effectively restricts tumor progression, and its combination with anti-PD-1 blockade exhibits strong synergy, maximizing CD8^+^ T cell infiltration and activation by simultaneously neutralizing the function of NOP14.

**Conclusion:**

Our findings establish NOP14 as a crucial oncogene and a key mediator of immune evasion in DLBCL that operates through the Wnt/β-catenin/c-Myc axis. Targeted NOP14 via NOP14-siRNA@LNP not only inhibits tumor growth but also sensitizes DLBCL to immune checkpoint blockade, offering a promising strategic framework for precision immunotherapy.

## 1 Introduction

Diffuse large B-cell lymphoma (DLBCL), an aggressive malignancy arising from mature B cells (1–3), represents the most common pathological subtype of non-Hodgkin lymphoma (NHL) (4, 5). Although the standard first-line rituximab-based immunochemotherapy regimen (R-CHOP) has significantly improved patient prognosis, 30% to 40% of patients eventually progress to relapsed or refractory (R/R) disease (6–9). In recent years, the clinical application of novel immunotherapies, such as chimeric antigen receptor T-cell (CAR-T) therapy, bispecific antibodies, and antibody-drug conjugates (ADCs), has revolutionized the treatment landscape for R/R DLBCL (10–13). Nonetheless, due to the profound biological heterogeneity of DLBCL (13, 14), a subset of patients still does not benefit from current treatments (15, 16). Therefore, discovering novel therapeutic targets and unraveling their underlying mechanisms are essential for advancing DLBCL treatment strategies.

NOP14 (Nucleolar Protein 14), a highly conserved factor in eukaryotes, primarily mediates 18S rRNA maturation and the biogenesis of the 40S ribosomal subunit (17). Recent studies have expanded our understanding of NOP14 functions, revealing its key regulatory role in the progression of various solid tumors (18–23). Specifically, in pancreatic cancer, NOP14 not only facilitates oncogenic signaling triggered by mutant p53 but also promotes tumor cell proliferation and migration by modulating key molecules such as MAPK3 and CDK2 (18, 19). In nasopharyngeal carcinoma, high NOP14 expression is significantly correlated with poor prognosis and has been confirmed to alter tumor sensitivity to rapamycin by activating the mTORC2-Akt signaling pathway (20). Emerging evidence also suggests that, in addition to promoting intrinsic malignant proliferation, NOP14 orchestrates the remodeling of the tumor microenvironment (21, 22). For instance, NOP14 expression level is positively associated with the infiltration of various immune cells, including CD8^+^ T cells, CD4^+^ T cells, NK cells, and dendritic cells, implying a potential immunomodulatory function in colon cancer (21). However, the biological function of NOP14 in DLBCL, particularly its influence on the immune landscape, remains largely unexplored.

In this study, we integrated bioinformatics analysis with experimental validation to systematically elucidate the role of NOP14 in DLBCL. Our data demonstrate that aberrant NOP14 upregulation correlates with unfavorable patient prognosis. Crucially, we uncover a dual functional identity of NOP14 in driving DLBCL progression by concurrently promoting cell-intrinsic proliferation and facilitating immune evasion. Mechanistically, NOP14 activates the Wnt/β-catenin/c-Myc signaling cascade to transcriptionally upregulate PD-L1 expression, thereby impairing CD8^+^ T cell infiltration and function. Additionally, we constructed a targeted lipid nanoparticle system (NOP14-siRNA@LNP) that not only effectively inhibits tumor growth but also exhibits potent synergistic efficacy with anti-PD-1 therapy. Collectively, our study establishes NOP14 as a promising therapeutic target and demonstrates that its inhibition significantly attenuates tumor progression, providing a novel strategy and theoretical basis for the clinical treatment of DLBCL.

## 2 Materials and methods

### 2.1 Patients and clinical samples

During the period from June 2024 to October 2025, a total of 60 newly diagnosed DLBCL samples and 60 reactive lymphoid tissue controls (from lymphadenitis patients) were collected at Qilu Hospital, Shandong University. Written informed consent was obtained from all participants. This study was conducted in accordance with the Declaration of Helsinki and its later amendments, and was approved by the Ethics Committee of Qilu Hospital, Shandong University (No. KYLL-2024(ZM)-161).

### 2.2 Prognostic and clinical statistical analysis

Kaplan-Meier survival curves were generated using the “survival” package in R, and the log-rank test was employed to compare differences in overall survival (OS) among DLBCL patients stratified by NOP14 expression status. Furthermore, the correlation between NOP14 expression levels and various clinical parameters was assessed. To determine whether NOP14 holds independent prognostic value, univariate and multivariate Cox proportional hazards regression analyses were subsequently performed. Visualization of all analytical results was accomplished using the “ggplot2” and “survminer” packages.

### 2.3 Construction of the NOP14-associated nomogram

Utilizing the “rms” package in R, a prognostic nomogram integrating NOP14 expression levels and clinicopathological factors was constructed to predict the probability of 1-, 3-, and 5-year survival. To evaluate the predictive performance of this nomogram, a calibration curve analysis was first conducted using the calibrate function to examine the consistency between predicted survival probabilities and actual observed outcomes. Furthermore, time-dependent receiver operating characteristic (ROC) curves were plotted, and the corresponding areas under the curve (AUC) at 1, 3, and 5 years were calculated to assess the model’s discriminatory ability. Finally, decision curve analysis (DCA) was applied to evaluate the clinical utility of the nomogram by comparing its net benefit against those of alternative predictors or strategies.

### 2.4 Cell culture

Human DLBCL cell lines, including GCB subtypes (DB, Pfeiffer, and SU-DHL-4) and non-GCB subtypes (OCI-LY3, and SU-DHL-2), along with the murine B-lymphoma A20 cell, were purchased from the ATCC. The U2932 cell line was purchased from the German Collection of Microorganisms and Cell Cultures. The HEK293T cell line was obtained from the Institute of Hematology and Blood Diseases Hospital, Chinese Academy of Medical Sciences and Peking Union Medical College and cultured in complete Dulbecco’s modified Eagle Medium (11965118, Gibco). All DLBCL cells were maintained in RPMI-1640 medium (11875119, Gibco) supplemented with 10% fetal bovine serum (S711-001S, Lonsera) and 1% penicillin-streptomycin (P1400, Solarbio) at 37 °C in a humidified incubator containing 5% CO_2_.

### 2.5 RNA isolation and qRT-PCR

Total RNA was extracted using TRIzol reagent (AG21101, Accurate Biology), followed by precipitation with trichloromethane, isopropanol, and absolute ethanol. The isolated RNA was dissolved in DEPC-treated water and its concentration was measured using a spectrophotometer. Subsequently, reverse transcription was performed using the Evo M-MLV Mix according to the manufacturer’s instructions (AG11706, Accurate Biology). Quantitative PCR (qPCR) was conducted on a Roche PCR amplification system using SYBR Green Premix Pro Taq (AG11701, Accurate Biology). The relative mRNA expression levels of target genes were normalized to GAPDH and calculated using the 2^-ΔΔCt^ method. The primer sequences used in this study are provided in **Supplementary Table 1**.

### 2.6 Western blotting

Total protein was extracted using M-PER™ Mammalian Protein Extraction Reagent (78501, Thermo Fisher Scientific) supplemented with protease and phosphatase inhibitors on ice. Protein samples were separated by SDS-PAGE and transferred onto PVDF membranes (IPVH00010, Millipore). After blocking with 5% non-fat milk (D8341, Solarbio) for 1 hour at room temperature, the membranes were incubated overnight at 4 °C with primary antibodies against NOP14 (26854-1-AP, Proteintech), GAPDH (60004-1-Ig, Proteintech), c-MYC (Proteintech, 67447-1-Ig), CCND1 (abcam, ab226977), PD-L1 (Proteintech, 66248-1-Ig), β-catenin (abcam, ab6302) and Actin (Abways, AB0035). The following day, the membranes were incubated with the corresponding secondary antibodies for 1 hour at room temperature. Protein bands were visualized using enhanced chemiluminescence (ECL) reagents (34094, Thermo Fisher Scientific) and captured with a digital imaging system (Tanon 5200).

### 2.7 Immunohistochemistry

Clinical specimens of paraffin-embedded human DLBCL tissues (obtained from the Department of Pathology, Qilu Hospital) and murine tumor sections were subjected to IHC staining. Briefly, the sections were deparaffinized and rehydrated through a graded ethanol series. Heat-induced antigen retrieval was performed in 0.01 M sodium citrate buffer (pH 6.0) at 95 °C for 15 minutes. To eliminate endogenous peroxidase activity, the slides were treated with 3% H_2_O_2_ and subsequently blocked with 5% normal goat serum for 1 hour at room temperature to reduce non-specific background staining. The sections were then incubated overnight at 4 °C with primary antibodies against NOP14 and Ki-67. On the following day, the sections were incubated with species-matched secondary antibodies for 1 hour at room temperature. Immunoreactivity was visualized using diaminobenzidine (DAB) substrate kit, followed by hematoxylin counterstaining. After dehydration and mounting with resin, images were captured via light microscopy and quantified using ImageJ software.

### 2.8 Plasmid construction and lentivirus transduction

The lentiviral shRNA plasmid targeting human and mouse NOP14 (shNOP14) and the lentiviral MYC plasmid targeting mouse were purchased from GeneChem (Shanghai, China). To produce lentiviral particles, the shRNA plasmids were co-transfected into HEK293T cells with the packaging plasmids psPAX2 and pMD2G using Lipofectamine 2000 (11668019, Invitrogen). Virus-containing supernatants were harvested at 48 and 72 hours post-transfection and subsequently filtered through a 0.45 μm pore-size membrane. For transduction, target cells were infected with the viral supernatant diluted in RPMI-1640 medium. After 24–48 hours of incubation, stable cell lines were selected using puromycin to ensure consistent shRNA expression. The specific shRNA sequences used in this study are provided in **Supplementary Table 2**.

### 2.9 Cell proliferation, colony formation and EdU assay

Cell viability was assessed using the CCK-8 kit (Vazyme Biotech, Nanjing, China). Briefly, cells were seeded into 96-well plates at a density of 3000 cells per well. Proliferation was monitored every 24 hours over a 4 days. At each time point, 10 μL of CCK-8 solution was added to each well, followed by a 4-hour incubation at 37 °C. The absorbance was then measured at 450 nm and 630 nm to determine the optical density (OD) values.

For the colony formation assay, cells (2000 cells/well) were inoculated into 24-well plates using methylcellulose medium (MethoCult™ H4230, Stemcell Technologies). Each experimental group was performed in triplicate. After two weeks, the resulting colonies were quantified using ImageJ software.

For EdU assay, cells were seeded in 12-well plates and incubated with 10 μM EdU for 2 hours. Subsequently, cells underwent fixation and staining according to the protocol provided by RiboBio (Guangzhou, China). Representative fluorescent images were captured using EVOS M5000 fluorescent microscope to ensure high-resolution visualization.

### 2.10 Establishment of mouse models

Six-to-eight-week-old BALB/c and NOG mice were obtained from Charles River and housed in a specific pathogen-free (SPF) environment. To establish the DLBCL mouse models, BALB/c and NOG mice were subcutaneously injected with 5 × 10^6^ A20 cells stably transfected with the respective viruses. Mice were euthanized 21 days post-inoculation, and tumor tissues were harvested to analyze tumor volume, weight, and immune cell infiltration, thereby evaluating the *in vivo* functional roles of NOP14 or MYC.

To evaluate the anti-tumor effect of NOP14-siRNA@LNP, DLBCL mouse models were established using A20 cells. The mice were intravenously injected with 1 mg/kg NOP14-siRNA@LNP on days 7, 9, and 11. On day 13, mice were anesthetized with isoflurane, and whole-body bioluminescence imaging was performed using the IVIS imaging system. Finally, tumors were harvested, weighed, and measured.

### 2.11 RNA sequencing analyses

Tumor tissues were harvested from an immunocompetent DLBCL mouse model and subjected to RNA sequencing (RNA-seq) for a comparative transcriptomic analysis between the NOP14-deficient (shNOP14) group and the control (shCtrl) group. The sequencing libraries were prepared and sequenced on the Illumina NovaSeq™ 6000 platform (LC SCIENCES, Hangzhou, China). Differentially expressed genes (DEGs) between groups were statistically screened using the edgeR package. Criteria for DEG identification were established as a q−value <0.05 and |log_2_(fold change)|≥1. Finally, Kyoto Encyclopedia of Genes and Genomes (KEGG) gene enrichment analysis was performed using DAVID software (https://davidbioinformatics.nih.gov/).

### 2.12 Flow cytometry analysis of tumor-infiltrating immune cells

Tumors were harvested and dissociated into single-cell suspensions. For surface staining, cells were incubated with specific antibodies at 4 °C for 30 min in the dark, followed by washing with PBS. For intracellular staining, cells were stimulated with 1× PMA/Ionomycin and BFA/Monensin (Multi Sciences, Hangzhou, China) for 4 h at 37 °C. After surface staining, fixation and permeabilization were performed using the Fix & Perm Kit (Multi Sciences) according to the manufacturer’s instructions, followed by intracellular cytokine labeling. Samples were analyzed via flow cytometry. The following antibodies were used in this study: eBioscience™ Fixable Viability Dye eFluor™ 780 (Thermo Scientific, 65-0865-14), PE/Cyanine7 anti-mouse CD8α (100722, BioLegend), APC anti-mouse CD4 (100516, BioLegend), PE/Cyanine7 anti-mouse CD11b (101216, BioLegend), FITC anti-mouse F4/80 (123108, BioLegend), PerCP/Cyanine5.5 anti-mouse CD49b (103520, BioLegend), APC anti-mouse Gr-1 (108412, BioLegend), APC anti-mouse IFN-γ (505809, BioLegend).

### 2.13 Construction of lipid nanoparticle

The lipid mixture consisted of SM-102, DSPC, cholesterol, and DMG-PEG2000+DSPE-PEG-cRGD (50:10:38.5:1.5). To calculate encapsulation efficiency, we measured the nucleic acid content difference between control and Triton X-100-treated LNPs. The final nucleic acid concentrations for the two formulations were 157.56 ng/μL and 164.13 ng/μL, respectively. Physicochemical characterization via DLS and TEM revealed a particle size of approximately 160–170 nm (PDI ~0.2) and a high encapsulation efficiency of 99%. For storage, the LNPs were kept in a 10 mM PBS solution supplemented with 10% sucrose at 2-8 °C in the dark.

### 2.14 Immunofluorescence analysis of CD8^+^ T cell infiltration

Tumor tissue samples were fixed, permeabilized, and blocked prior to incubation with anti-CD8α primary antibody (ab217344, Abcam) overnight at 4 °C. Subsequently, cells were incubated with an Alexa Fluor 594-conjugated secondary antibody for 1 h at room temperature protected from light. Samples were mounted with DAPI-containing anti-fade mounting medium. Images were captured using EVOS M5000 fluorescent microscope, and data analysis was performed using ImageJ.

### 2.15 Statistical analysis

To validate data reliability and reproducibility, all biological replicates were performed independently at least three times. Quantitative results were expressed as the mean ± standard deviation (SD). Computational processing and statistical evaluations were executed using R (version 4.3.3), GraphPad Prism 9, and Perl scripts. A *p* value of less than 0.05 was established as the threshold for statistical significance across all analyses.

## 3 Results

### 3.1 NOP14 is upregulated and correlates with poor prognosis in DLBCL

**Figure 1:**
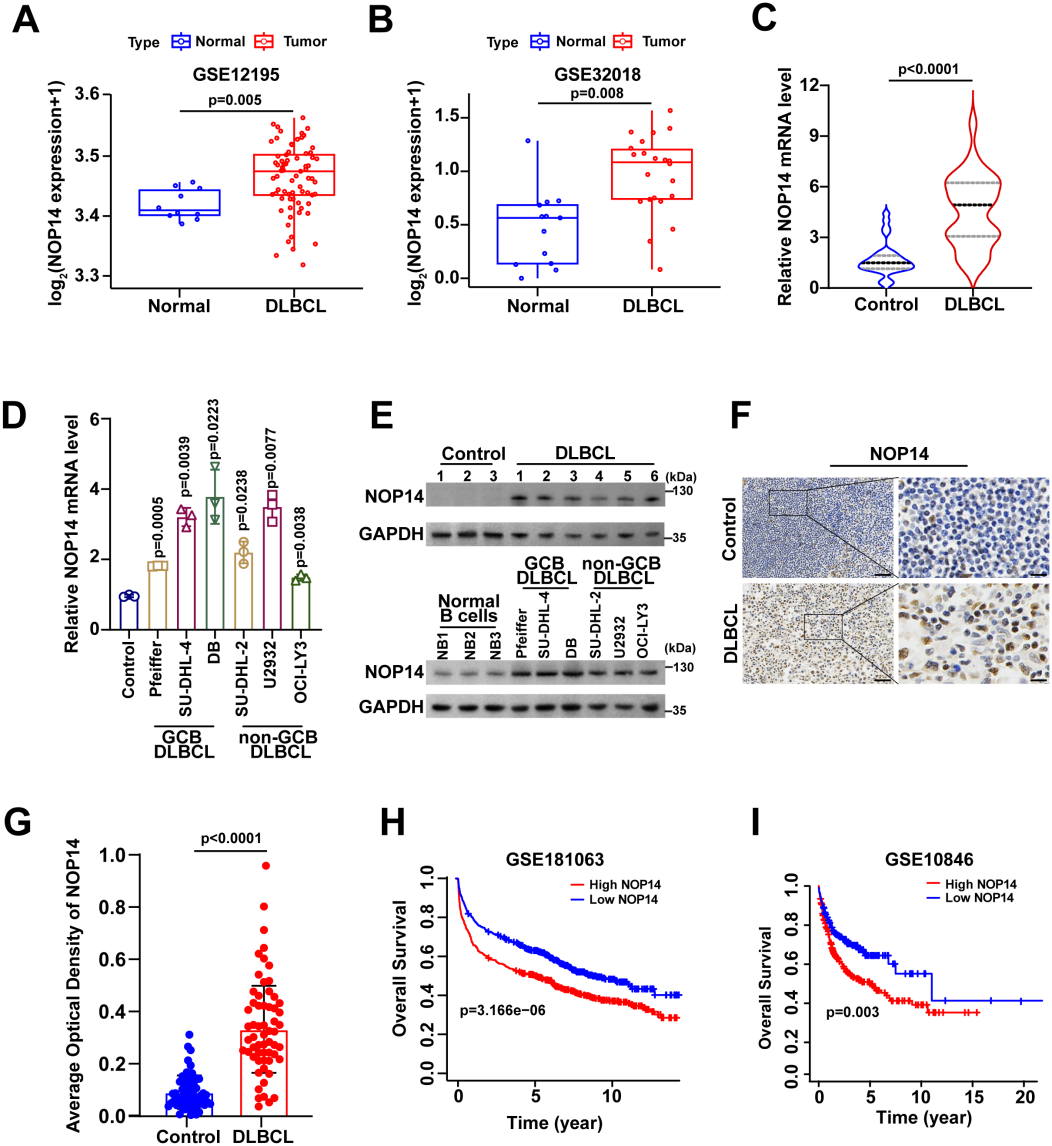
NOP14 is upregulated and correlates with poor prognosis in DLBCL. **(A, B)** Analysis of NOP14 mRNA expression levels in DLBCL tissues compared to normal tissues using the GEO datasets GSE12195 **(A)** and GSE32018 **(B)**. **(C)** qRT-PCR analysis was used to assess the relative NOP14 mRNA levels in clinical DLBCL samples (n=30) and reactive lymphoid tissues (n=30). **(D)** Relative NOP14 mRNA expression levels in normal B cells compared with various human DLBCL cell lines categorized into germinal center B-cell-like (GCB) and non-GCB subtypes. **(E)** Western blot analysis showed NOP14 protein expression in DLBCL clinical tissues (top panel) and cell lines (bottom panel). **(F, G)** Representative micrographs of immunohistochemical (IHC) staining **(F)** and the corresponding statistical quantification of the average optical density **(G)** of NOP14 protein expression in reactive lymphoid control tissues (n =60) and DLBCL tissues (n=60) Scale bar:50 μm (left); 12.5 μm (right). **(H, I)** Kaplan-Meier overall survival (OS) curves comparing DLBCL patients with high versus low NOP14 expression based on data from GSE181063 **(H)** and GSE10846 **(I)** cohorts. Statistical significance was defined as p <0.05. For A, B, C and G, data are analyzed by two-tailed unpaired Student’s *t* test. For D, data are analyzed by two-tailed paired Student’s *t* test. For H and I, data are analyzed by log-rank (Mantel-Cox) test.

To investigate the potential role of NOP14 in DLBCL, we first analyzed the GSE12195 and GSE32018 datasets, which identified a significant upregulation of NOP14 mRNA expression in DLBCL tissues compared with normal controls (**Figures 1A, B**). This finding was validated via qRT-PCR and Western blot assays, confirming robust NOP14 overexpression in both clinical specimens and DLBCL cell lines (**Figures 1C–E**). Consistently, immunohistochemical (IHC) staining visualized the aberrant accumulation of NOP14 protein in tumor tissues, with significantly higher optical density observed in DLBCL samples than in reactive lymphoid tissues (**Figures 1F, G**). Furthermore, survival analyses of the GSE181063 and GSE10846 cohorts revealed that high NOP14 expression was associated with significantly reduced overall survival (**Figures 1H, I**). Collectively, our findings demonstrate that NOP14 is aberrantly overexpressed in DLBCL and correlates with unfavorable clinical outcomes, suggesting its potential role as an oncogene.

### 3.2 Elevated NOP14 expression correlates with adverse clinicopathological features and enhances prognostic nomogram performance

**Figure 2:**
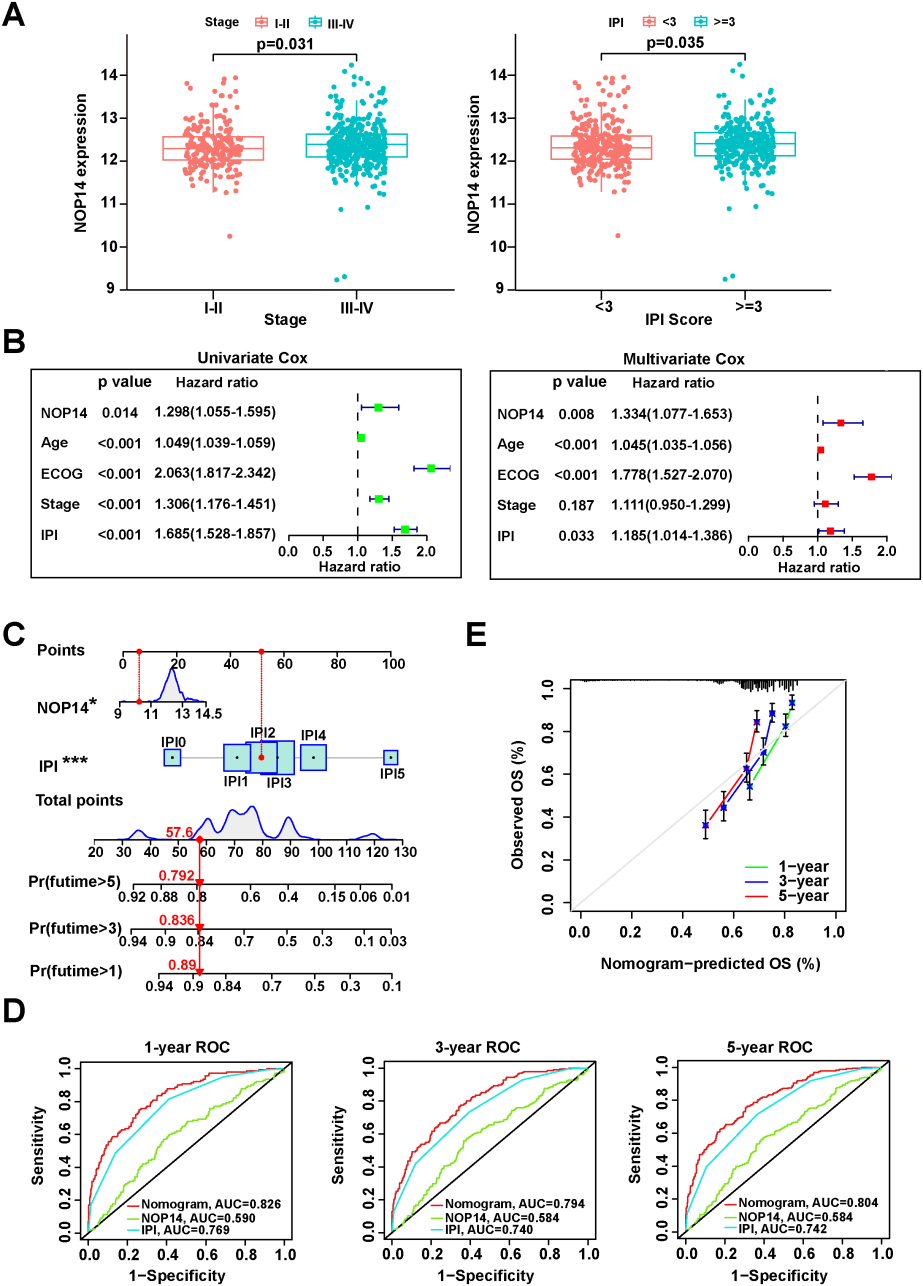
Elevated NOP14 expression correlates with adverse clinicopathological features and enhances prognostic nomogram performance. **(A)** Box plots showing the relationship between NOP14 expression levels and key clinical characteristics, including Ann Arbor stage (Stage I–II versus Stage III–IV, left panel) and International Prognostic Index score (IPI< 3 versus IPI ≥ 3, right panel), analyzed within the GSE10846 database. **(B)** Univariate (left panel) and multivariate (right panel) Cox regression analyses evaluating the independent prognostic significance of NOP14 expression alongside conventional clinical variables, including Age, ECOG, Stage, and IPI score, in the GSE10846 dataset. **(C)** A prognostic nomogram integrated with NOP14 expression levels and IPI scores to predict the 1-year, 3-year, and 5-year overall survival (OS) probabilities of DLBCL patients derived from the GSE10846 cohort. **(D)** ROC curves comparing the predictive accuracy (AUC) of the nomogram, NOP14 expression alone, and IPI score alone for 1-, 3-, and 5-year overall survival. **(E)** Calibration curves evaluating the consistency between the nomogram-predicted OS probabilities and the actually observed OS rates at 1-year (green line), 3-year (blue line), and 5-year (red line) landmarks, where the light grey 45-degree line represents an ideal predictive reference. Statistical significance was defined as p <0.05.

To elucidate the clinical significance of NOP14, we conducted logistic regression analysis to examine its correlation with clinicopathological characteristics in DLBCL. The results demonstrated that in the GSE10846 cohort, NOP14 expression was significantly elevated in patients with advanced disease stages (III-IV) and higher International Prognostic Index scores (IPI ≥3), suggesting an association with aggressive disease progression (**Figure 2A**). Both univariate and multivariate Cox regression analyses identified NOP14 as an independent prognostic factor for overall survival (**Figure 2B**). To facilitate clinical application, we constructed a prognostic nomogram integrating NOP14 expression and IPI scores to predict 1-, 3-, and 5-year survival probabilities (**Figure 2C**). Notably, this nomogram outperformed both the IPI score and NOP14 alone, achieving superior predictive accuracy with 1-, 3-, and 5-year AUCs of 0.826, 0.794, and 0.804, respectively (**Figure 2D**). Calibration curves further demonstrated high consistency between the nomogram-predicted and the actual observed survival rates (**Figure 2E**). Together, these results highlight the robust predictive value of the NOP14-incorporated nomogram for DLBCL patient stratification.

### 3.3 Knockdown of NOP14 inhibits DLBCL cell proliferation *in vitro*

**Figure 3:**
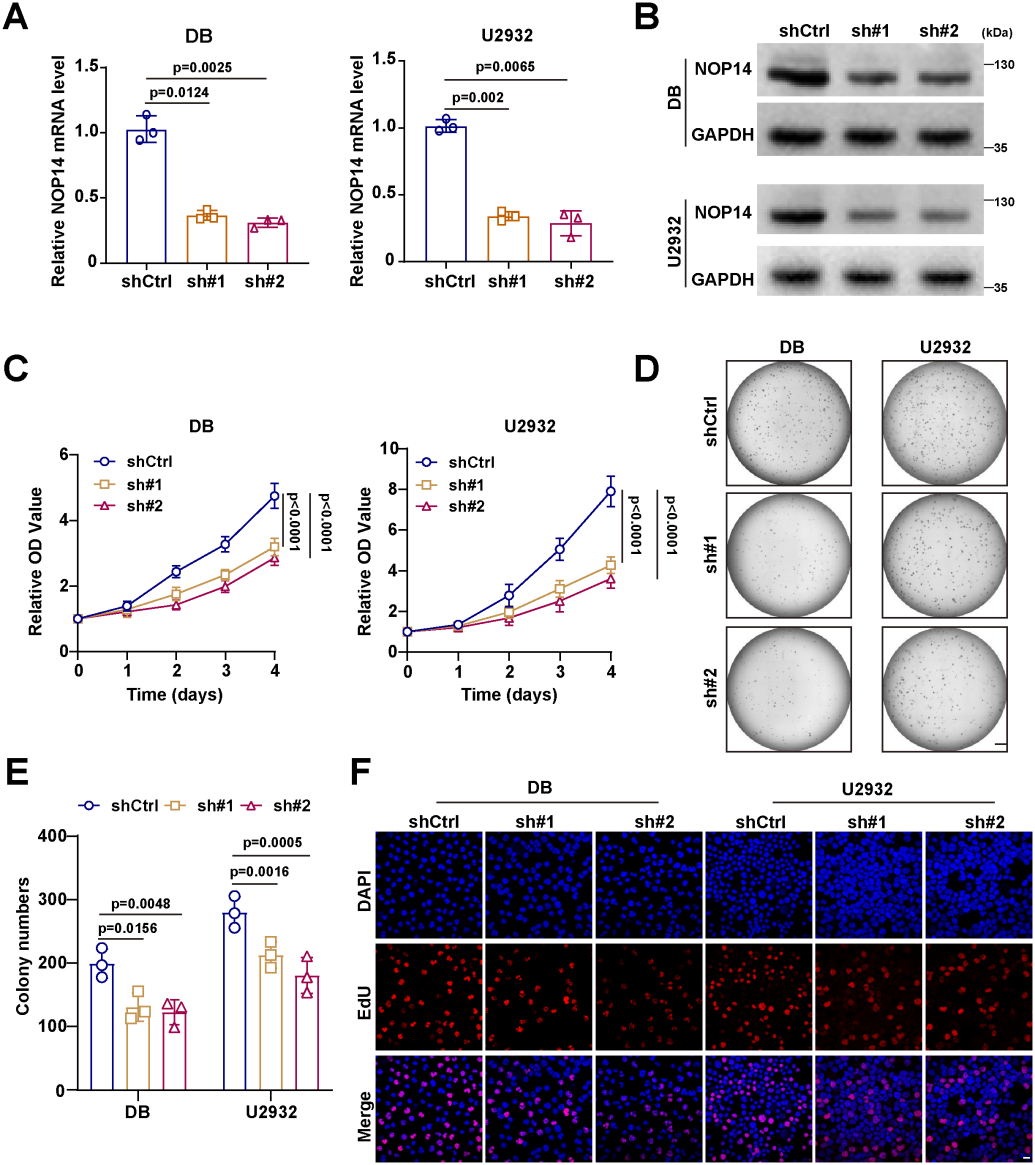
Knockdown of NOP14 inhibits DLBCL cell proliferation *in vitro*. **(A, B)** qRT-PCR and Western blot analyses were performed to verify the knockdown efficiency of NOP14 at both mRNA and protein levels in DB and U2932 cells (30000 cells/mL) transfected with control short hairpin RNA (shCtrl) or two independent NOP14-targeting sequences (sh#1 and sh#2). **(C)** CCK-8 assays showing the proliferation rates of control and NOP14-knockdown cells over time. **(D, E)** Representative images and corresponding statistical quantification of colony-forming ability in DB and U2932 cells transfected with shCtrl or shNOP14. **(F)** Representative fluorescence micrographs of EdU incorporation assays evaluating the proliferation capacity of DB and U2932 cell lines transfected with shCtrl or shNOP14. Scale bar: 10 μm. Statistical significance was defined as p <0.05. For **(A, E)**, data are analyzed by one-way ANOVA. For **(C)**, data are analyzed by two-way ANOVA.

To investigate the biological function of NOP14 in DLBCL, we established stable NOP14 knockdown models in GCB-subtype DB and non-GCB-subtype U2932 cell lines using lentiviral-mediated shRNAs. The silencing efficiency was verified at both mRNA and protein levels via qRT-PCR and Western blotting (**Figures 3A, B**). Functionally, knockdown of NOP14 significantly compromised cell viability and clonogenic potential, as evidenced by the CCK-8 and colony formation assays (**Figures 3C–E**). Consistently, EdU incorporation assays revealed a marked reduction in DNA synthesis following NOP14 depletion (**Figure 3F**). In conclusion, these findings demonstrate that NOP14 promotes the proliferation of DLBCL cells *in vitro*.

### 3.4 NOP14 facilitates DLBCL progression *in vivo*

**Figure 4:**
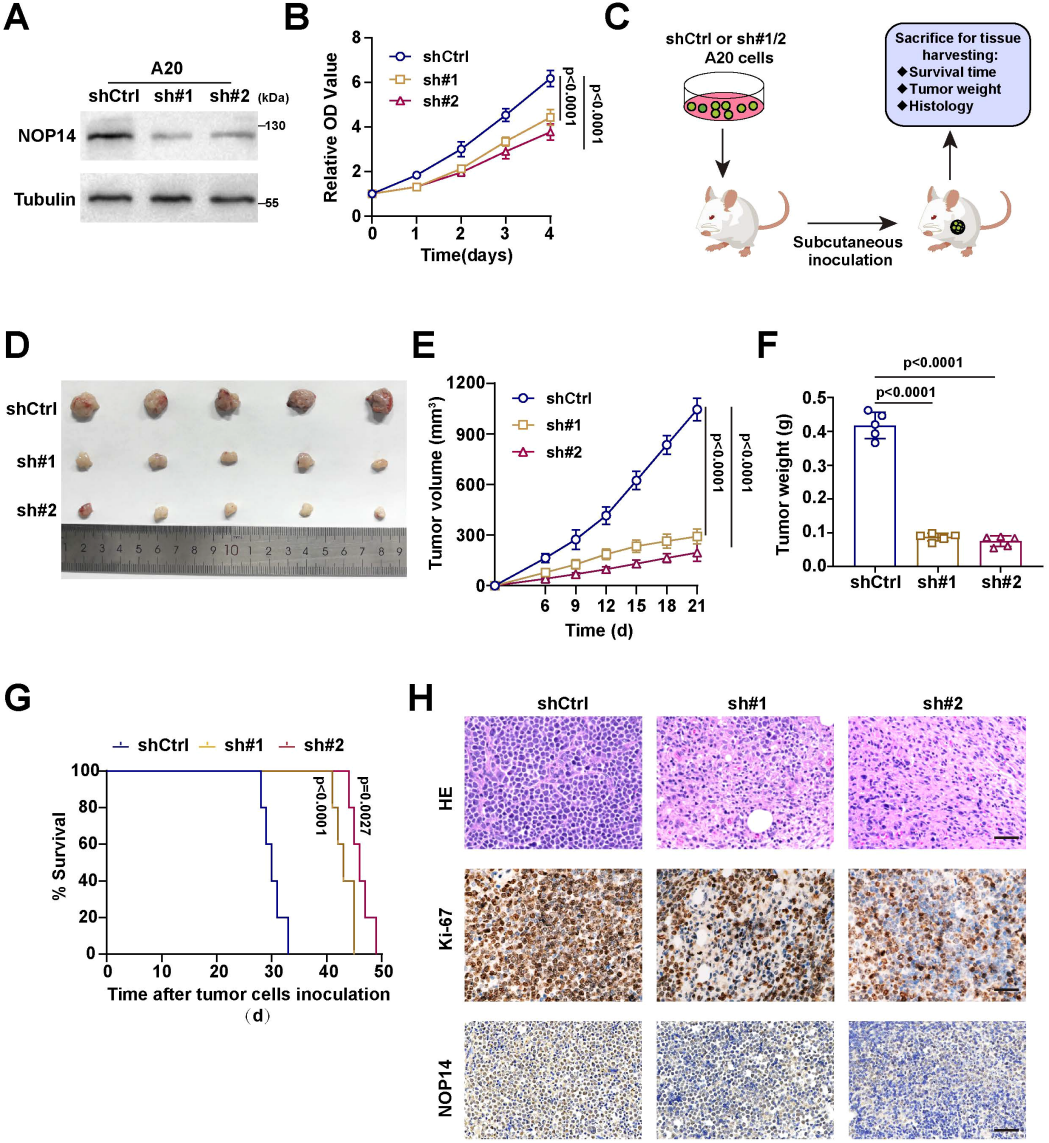
NOP14 facilitates DLBCL progression *in vivo.* **(A)** Western blot analysis validation of NOP14 knockdown efficiency in murine A20 cells stably transfected with shCtrl or shNOP14. **(B)** CCK-8 analysis showed the time-dependent cell proliferation curves of A20 cells in shCtrl, sh#1 and sh#2 groups (30000 cells/mL). **(C)** Schematic workflow illustrating the experimental design of the syngeneic lymphoma mouse model, in which BALB/c mice were subcutaneously inoculated with shCtrl- or shNOP14-expressing A20 cells (5×10^6^ cells per mouse) followed by subsequent monitoring of survival time, tumor weight, and histological alterations. **(D-F)** Assessment of *in vivo* tumor growth and burden in BALB/c mice subcutaneously inoculated with shCtrl, sh#1, or sh#2-expressing A20 cells (n=5 per group), showing representative images of harvested tumors at day 21 **(D)**, dynamic tumor volume growth curves measured over a 21-day time course **(E)**, and the corresponding statistical quantification of terminal tumor weights at sacrifice **(F)**. **(G)** Kaplan-Meier curves analyzing the percentage of overall survival in tumor-bearing mice (n=5 per group) over the designated time course following initial tumor cell inoculation. **(H)** Representative histological and immunohistochemical micrographs of harvested tumor tissues sections subjected to hematoxylin and eosin (HE) staining alongside IHC evaluation for the proliferation marker Ki-67 and target protein NOP14. Scale bar: 50 μm. Statistical significance was defined as p <0.05. For **(B, E)**, data are analyzed by two-way ANOVA. For **(F)**, data are analyzed by one-way ANOVA. For **(G)**, data are analyzed by log-rank (Mantel-Cox) test.

To corroborate the oncogenic function of NOP14 *in vivo*, we initially knocked down NOP14 in murine A20 lymphoma cells and confirmed that its downregulation significantly inhibited A20 cell proliferation *in vitro* (**Figures 4A, B**). Subsequently, these cells were subcutaneously inoculated into mice to successfully establish a syngeneic mouse lymphoma model (**Figure 4C**). Consistent with our *in vitro* data, NOP14 silencing significantly attenuated lymphoma progression, as evidenced by a marked reduction in both tumor volume and weight compared to the control group (**Figures 4D–F**). Furthermore, mice bearing NOP14-deficient tumors exhibited significantly prolonged overall survival (**Figure 4G**). Histopathological examination indicated that NOP14 knockdown resulted in reduced tumor infiltration (H&E), while Ki-67 immunostaining confirmed a substantial decrease in the proliferation of tumor cells (**Figure 4H**). Collectively, these findings suggest that NOP14 is essential for lymphoma progression *in vivo*.

### 3.5 NOP14 knockdown in tumor cells enhances CD8^+^ T cell-mediated anti-tumor immunity

**Figure 5:**
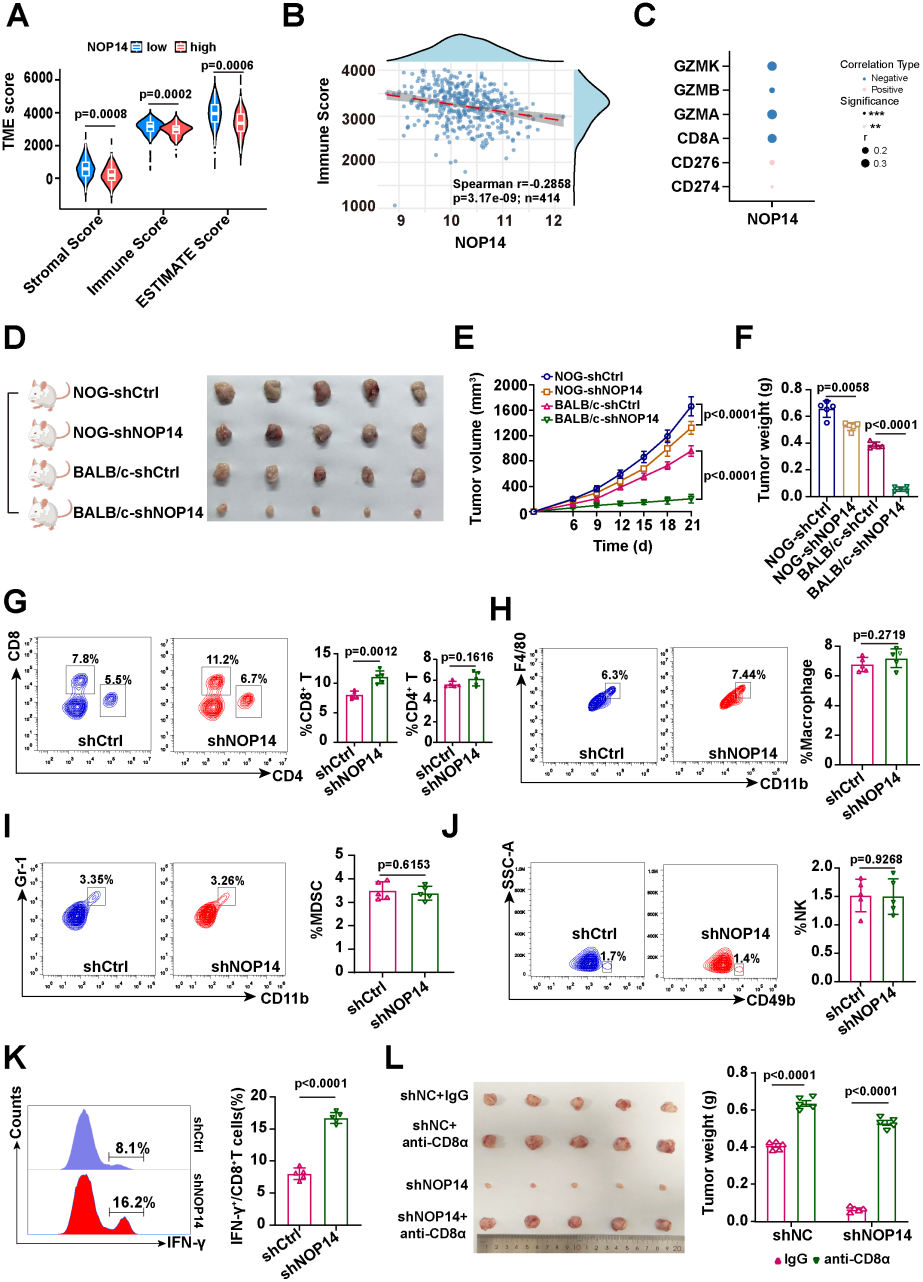
NOP14 knockdown in tumor cells enhances CD8^+^ T cell-mediated anti-tumor immunity. **(A)** Violin plots comparing TME scores, including StromalScore, ImmuneScore, and ESTIMATEscore, between the NOP14-low and NOP14-high expression groups within the GSE10846 cohort calculated via the ESTIMATE algorithm. **(B)** Spearman correlation analysis exhibiting the significant negative correlation between NOP14 mRNA expression and the calculated ImmuneScore in the GSE10846 dataset. **(C)** Bubble plot illustrating the correlation between NOP14 expression and core immune-related markers, including cytotoxic molecules (GZMK, GZMB, GZMA), T cell surface markers (CD8A), and immune checkpoints (CD276, CD274), where blue and pink circles represent negative and positive correlations, respectively, and circle sizes indicate the absolute values of the Spearman correlation coefficient (r). **(D-F)** Comparative assessment of *in vivo* tumor growth and burden in immunodeficient NOG mice and immunocompetent BALB/c mice subcutaneously inoculated with shCtrl or shNOP14-transfected A20 cells (n=5 per group), showing photographs of completely harvested tumors at day 21 **(D)**, dynamic tumor volume growth curves measured over time **(E)**, and the corresponding statistical quantification of terminal tumor weights at sacrifice **(F)**. **(G-J)** Representative flow cytometric plots (left) and statistical percentage quantification (right) of major tumor-infiltrating immune cell populations isolated from harvested BALB/c mouse tumors (n=5 per group), including CD8^+^ T cells, CD4^+^ T cells, macrophages, MDSCs and NK cells (n=5 per group). **(K)** Representative flow cytometric histograms (left) and corresponding percentage quantification (right) of active intra-tumoral IFN-γ^+^ CD8^+^ T cells population in the shCtrl and shNOP14 groups. **(L)** Representative tumor images of harvested tumors (left) and corresponding statistical analysis of terminal tumor weights (right) evaluating shNC or shNOP14-derived tumors in BALB/c mice treated with either control IgG or anti-CD8α depleting antibodies (200 μg per mouse, n=5 per group).Statistical significance was defined as p <0.05. For **(E)**, data are analyzed by two-way ANOVA. For F, data are analyzed by one-way ANOVA. For **(G–L)**, data are analyzed by two-tailed unpaired Student’s *t* test.

Given the pivotal role of the tumor microenvironment (TME) in malignant progression, we further explored the correlation between NOP14 and the immune landscape. Bioinformatic profiling revealed that the Stromal Score, Immune Score, and ESTIMATE Score in the NOP14-high group were significantly lower than those in the NOP14-low group (**Figure 5A**), with NOP14 expression demonstrating a remarkably negative correlation with the Immune Score (**Figure 5B**). Further analyses indicated that NOP14 expression was significantly inversely correlated with the CD8^+^ T cell marker (CD8A) and core cytotoxic molecules (GZMA, GZMB, and GZMK), but displayed a distinct positive correlation with the immune checkpoint molecules CD276 and CD274 (PD-L1) (**Figure 5C**). These bioinformatic insights suggest that NOP14 in tumor cells may exert its pro-oncogenic functions by suppressing the immune infiltration and activity of CD8^+^ T cells. To validate this hypothesis, we established syngeneic tumor models in both immunodeficient (NOG) and immunocompetent (BALB/c) mice. While NOP14 knockdown inhibited tumor growth in both models, the suppression was roughly three-fold more pronounced in immunocompetent hosts than in their immunodeficient counterparts. (**Figures 5D–F**). This discrepancy implies that NOP14-driven tumorigenesis relies, at least in part, on the adaptive immune system.

Subsequently, we used flow cytometry to further analyze immune cell infiltration within the tumor microenvironment. Consistent with the bioinformatic predictions, NOP14 downregulation significantly increased the infiltration of CD8^+^ T cells, whereas no notable changes were detected in CD4^+^ T cells, MDSCs, macrophages, or NK cells (**Figures 5G–J**). To further clarify the functional impact of NOP14 on CD8^+^ T cells, we measured the level of its effector cytokine IFN−γ. As expected, NOP14 knockdown significantly enhanced IFN−γ production (**Figure 5K**), indicating the robust activation of CD8^+^ T cell cytotoxicity.

Finally, to confirm whether these CD8^+^ T cells are indispensable for the NOP14-mediated anti-tumor response, we specifically depleted CD8^+^ T cells in BALB/c mice using an anti-CD8α antibody prior to A20 cell inoculation. The results indicated that the tumor-suppressive effect induced by NOP14 knockdown was significantly attenuated upon the depletion of CD8^+^ T cells (**Figure 5L**). Notably, however, even after eliminating CD8^+^ T cell-mediated immunity, tumor growth in the shNOP14 group remained distinctly slower than that in the CD8 depletion group (**Figure 5L**).

In summary, these findings confirm that, in addition to directly driving tumor cell proliferation, NOP14 contributes to tumor progression by suppressing CD8^+^ T cell-mediated antitumor immunity.

### 3.6 NOP14 promotes DLBCL progression and immune evasion via the Wnt/β-catenin/c-Myc axis

**Figure 6:**
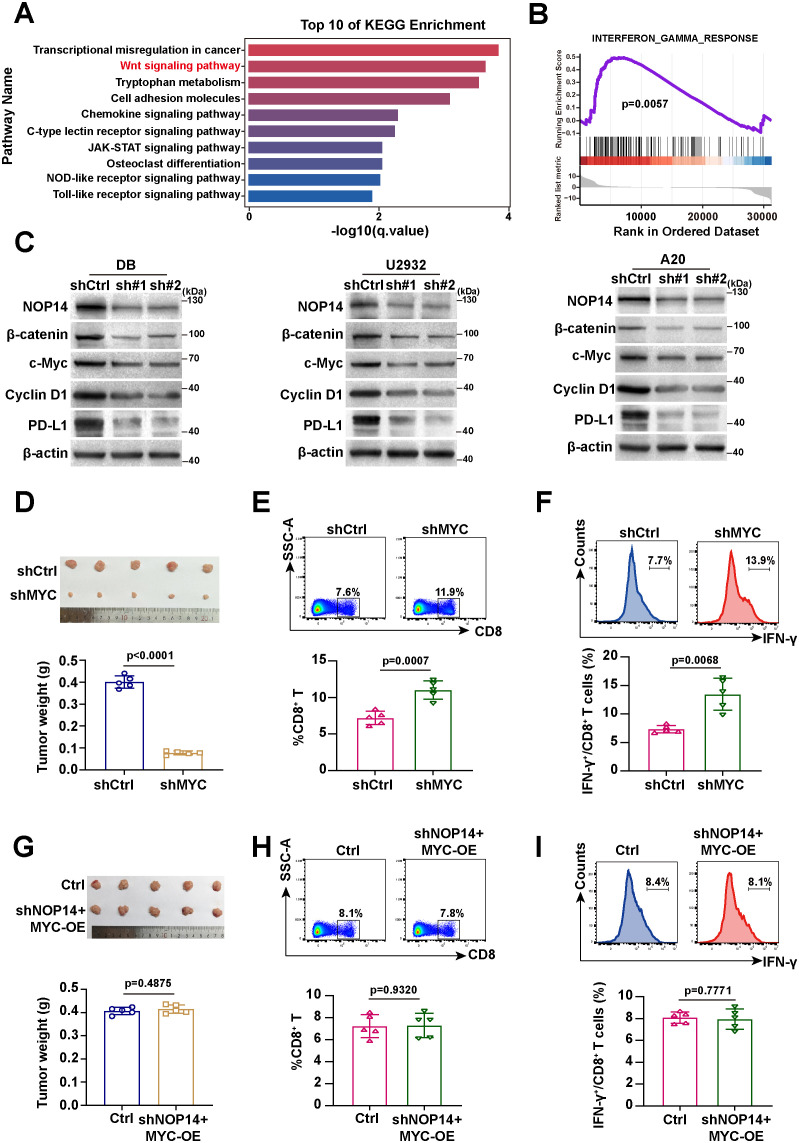
NOP14 promotes DLBCL progression and immune evasion via the Wnt/β-catenin/c-Myc axis. **(A)** KEGG pathway enrichment analysis showing the top 10 significantly enriched pathways, with the Wnt signaling pathway highlighted in red. Genes with a q value <0.05 and |log2(fold-change)|≥1 were considered differentially expressed genes (DEGs). **(B)** Gene Set Enrichment Analysis (GSEA) plot displaying the strong correlation between NOP14 expression and the activation of the “INTERFERON_GAMMA_RESPONSE” hallmark gene set. **(C)** Western blot analysis of NOP14, β-catenin, c-Myc, Cyclin D1, and PD-L1 protein levels in DB, U2932, and A20 cells transfected with shCtrl or shNOP14. **(D)** Representative images **(top)** and quantification of tumor weight (bottom) of subcutaneous tumors from A20-bearing BALB/c mice injected with shCtrl or shMYC cells (n=5 per group). **(E, F)** Flow cytometric analysis and corresponding quantification of tumor-infiltrating CD8^+^ T cells **(E)** and IFN-γ^+^ CD8^+^ T cells **(F)** in shCtrl and shMYC groups. **(G)** Representative photographs of harvested subcutaneous tumors (top) and corresponding statistical analysis of terminal tumor weights (bottom) from the *in vivo* rescue cohort, evaluating tumor-bearing BALB/c mice assigned to the Ctrl group and the shNOP14 + c-Myc overexpression (shNOP14 + MYC-OE) group (n=5 per group). **(H, I)** Flow cytometric analysis and quantification of tumor-infiltrating CD8^+^ T cells **(H)** and IFN-γ^+^ level **(I)** between the Ctrl and shNOP14 + MYC-OE groups. Statistical significance was defined as p <0.05. For **(D G)**, data are analyzed by one-way ANOVA. For **(E, F, H I)**, data are analyzed by two-tailed unpaired Student’s t test.

In light of our findings demonstrating the dual capacity of NOP14 to promote tumor proliferation and suppress CD8^+^ T cell infiltration and activation, we next sought to investigate the intrinsic molecular mechanisms within tumor cells that coordinate this aggressive growth and immunosuppressive microenvironment. Consequently, RNA sequencing (RNA-seq) was performed on tumor tissues harvested from immunocompetent DLBCL mouse models of the control and NOP14-knockdown (shNOP14) groups. KEGG pathway enrichment analysis identified the Wnt signaling pathway as a primary downstream target of NOP14 (**Figure 6A**). Furthermore, gene set enrichment analysis (GSEA) revealed that NOP14 downregulation was closely associated with the activation of the IFN-γ response pathway (**Figure 6B**).

Aberrant activation of the Wnt pathway is well known to trigger the stabilization and nuclear translocation of the core transcription factor β-catenin, which subsequently drives the transcription of the downstream proto-oncogene c-Myc to fuel tumor progression (24, 25). Meanwhile, accumulating evidence highlights c-Myc as a pivotal regulator of immune checkpoints that directly binds to and transcriptionally upregulates PD-L1 expression, which represents a classic mechanism through which tumor cells impair CD8^+^ T cell infiltration and blunt the IFN-γ response (26, 27). Based on these findings, we hypothesized that NOP14 fuels the Wnt/β-catenin/c-Myc signaling axis, which simultaneously drives intrinsic tumor proliferation and orchestrates immune escape via the upregulation of PD-L1. As we expected, western blot analysis confirmed that NOP14 knockdown resulted in a significant reduction in the protein levels of β-catenin, c-Myc and PD-L1 (**Figure 6C**), demonstrating that NOP14 expression potently activates the Wnt/β-catenin signaling cascade.

To ascertain whether the oncogenic and immunosuppressive effects of NOP14 are dependent on the Wnt/β-catenin/c-Myc axis, we first knocked down MYC (shMYC) in A20 cells and established a mouse lymphoma model (**Supplementary Figure 1A**). Our results showed that the knockdown of c-Myc substantially impeded tumor progression in DLBCL mice (**Figure 6D**). More importantly, flow cytometric analysis revealed a prominent elevation in the proportion of infiltrating CD8^+^ T cells within shMYC-derived tumors (**Figure 6E**). This was accompanied by a substantial increase in IFN-γ production from these tumor-infiltrating cells, which is highly consistent with the immunophenotype observed following NOP14 knockdown (**Figure 6F**). To further substantiate the functional dependency on this axis, we performed an *in vivo* rescue experiment by overexpressing c-Myc in NOP14-knockdown A20 cells (**Supplementary Figure 1B**). Remarkably, restoring c-Myc completely reversed the tumor growth restriction caused by NOP14 knockdown, bringing tumor weight back to control levels (**Figure 6G**). Consistently, the enhanced CD8^+^ T cell infiltration (**Figure 6H**) and IFN-γ production (**Figure 6I**) observed in NOP14-knockdown tumors were completely blocked after c-Myc re-expression.

In conclusion, these results indicate that NOP14 activates the Wnt/β-catenin/c-Myc pathway, thereby driving cell proliferation and sustaining an immunosuppressive microenvironment to promote DLBCL progression.

### 3.7 NOP14-siRNA@LNP enhances antitumor immunity and synergizes with PD-1 blockade

**Figure 7:**
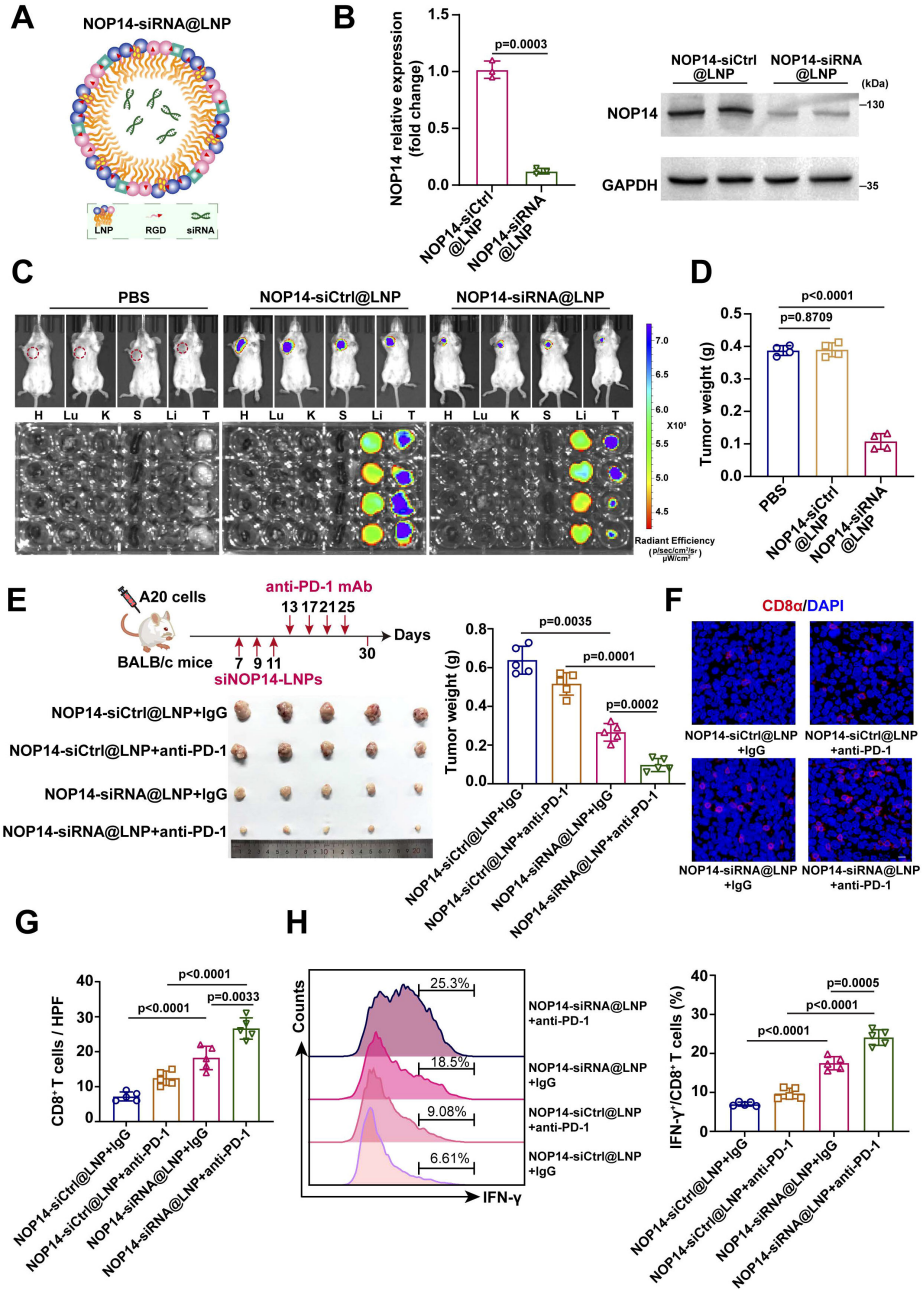
NOP14-siRNA@LNP enhances antitumor immunity and synergizes with PD-1 blockade. **(A)** Schematic illustration of the structure of NOP14-siRNA@LNP, which is encapsulated by a lipid nanoparticle (LNP) and surface-modified with RGD peptides for targeted delivery. **(B)** Relative mRNA expression (left) and protein levels (right) of NOP14 in A20 cells treated with 1 μg/mL siCtrl@LNP or NOP14-siRNA@LNP. **(C, D)** Representative *in vivo* fluorescence imaging and *ex vivo* tissue distribution (H: heart, Lu: lung, K: kidney, S: spleen, Li: liver, T: tumor) of mice treated with PBS, NOP14-siCtrl@LNP, or NOP14-siRNA@LNP **(C)**, and the corresponding statistical analysis of tumor weight **(D)** (n =4 per group). **(E)** Schematic protocol of the combination therapy in BALB/c mice bearing A20 tumors (top). Mice were treated with siNOP14-LNPs on days 7, 9, and 11, followed by 200 μg per mouse anti-PD-1 mAb or IgG isotype control injections on days 13, 17, 21, and 25. Representative photographs of excised tumors from each group on day 30 (bottom left) and quantitative analysis of tumor weights (bottom right). **(F, G)** Representative immunofluorescence **(IF)** staining images **(F)** and corresponding quantitative analysis **(G)** of tumor-infiltrating CD8^+^ T cells (red) in tumor tissues from the indicated treatment groups. Nuclei were counterstained with DAPI (blue). Scale bar:10 μm. **(H)** Flow cytometry analysis (left) and quantification (right) of the percentage of IFN-γ^+^ producing CD8^+^ T cells in tumor-infiltrating lymphocytes from different treatment groups. Statistical significance was defined as p <0.05. For **(B)**, data are analyzed by two-tailed paired Student’s *t* test. For **(D, E, G H)**, data are analyzed by one-way ANOVA.

Our findings demonstrate that NOP14 inhibition effectively suppresses tumor progression, identifying NOP14 as a promising therapeutic target for DLBCL. Based on this, we developed cRGD-modified lipid nanoparticles (LNPs) encapsulated with NOP14-siRNA (NOP14-siRNA@LNP) to facilitate specific targeting of DLBCL cells via αvβ3 integrin recognition (28, 29) (**Figure 7A**). Characterization of these LNPs revealed a uniform particle size distribution approximately 160–170 nm (**Supplementary Figures 2A, B**). The knockdown efficiency was validated in A20 cells using qRT-PCR and western blot analyses, which confirmed that NOP14-siRNA@LNP significantly reduced NOP14 expression at both the mRNA and protein levels compared to the control group (siCtrl@LNP) (**Figure 7B**).

To evaluate the *in vivo* delivery efficiency of the LNPs, an A20 syngeneic mouse model was established. Following multiple intravenous injections of the LNPs, *in vivo imaging system* (IVIS) analysis revealed that NOP14-siRNA@LNPs predominantly accumulated at the tumor site, followed by fluorescence observed in the liver (**Figure 7C**). This targeted enrichment confirms that cRGD modification effectively mediates the directional delivery of NOP14-siRNA@LNPs to DLBCL cells *in vivo*. Crucially, evaluation of tumor weight revealed that NOP14-siRNA@LNPs markedly impeded tumor progression (**Figure 7D**). Meanwhile, toxicity evaluations of key organs showed that the nanoparticles possessed a favorable safety (**Supplementary Figure 3**).

Given the role of NOP14 in modulating the immune microenvironment, we also evaluated its synergy with the immune checkpoint inhibitor anti-PD-1. As hypothesized, NOP14-siRNA@LNP monotherapy significantly inhibited tumor progression in lymphoma-bearing mice, while the combination of NOP14-siRNA@LNP and anti-PD-1 exerted a markedly more potent inhibitory effect (**Figure 7E**). Immunofluorescence staining demonstrated that NOP14-siRNA@LNP treatment increased the infiltration of CD8^+^ T cells within the tumor tissue, which was further amplified in the combination therapy group (**Figures 7F, G**). Furthermore, flow cytometric analysis showed that the expression of the effector cytokine IFN-γ was upregulated following NOP14 knockdown and reached its highest levels under the combined treatment regimen (**Figure 7H**). Collectively, these results demonstrate that nanoparticle-mediated NOP14 inhibition activates antitumor immune responses and significantly sensitizes DLBCL to immune checkpoint blockade, offering a novel strategic framework for DLBCL immunotherapy.

## 4 Discussion

Despite advancements in therapeutic modalities, including chemotherapy and immunotherapy, relapsed or refractory (R/R) disease remains a formidable clinical challenge in DLBCL, primarily driven by profound tumor heterogeneity (30–32). In the present study, we identified NOP14 as a pivotal oncogenic driver and a compelling therapeutic target in DLBCL. Consistent with prior reports implicating the oncogenic role of NOP14 in pancreatic cancer and nasopharyngeal carcinoma (18–20), our data demonstrate that NOP14 is aberrantly upregulated in DLBCL tissues and cell lines. Beyond mere expression profiling, our study also highlights the significant clinical implications of NOP14. We confirmed that elevated NOP14 expression serves as an independent prognostic factor, closely correlating with advanced disease stage and higher IPI scores. Notably, a prognostic nomogram integrating NOP14 expression with IPI scores exhibited superior predictive performance compared to conventional metrics alone. These findings suggest that NOP14 is not just involved in tumorigenesis but is a valuable biomarker that could refine risk stratification for DLBCL patients.

The complex immune landscape of the tumor microenvironment is a decisive factor in DLBCL progression and therapeutic response (33–36). Antitumor activity of CD8^+^ T lymphocytes is frequently hampered by immunosuppressive subsets, such as regulatory T cells (Tregs), myeloid-derived suppressor cells (MDSCs), or tumor-associated macrophages (TAMs), facilitating immune evasion (37–41). Our study reveals that NOP14 drives intrinsic malignant proliferation while simultaneously orchestrating an immunosuppressive TME. Comparative experiments in immunocompetent versus immunodeficient mice provided robust evidence that NOP14-mediated tumor progression is partially dependent on the adaptive immune system. Mechanistically, NOP14 downregulation represses the Wnt/β-catenin/c-Myc signaling axis, which subsequently modulates PD-L1 expression, enhances CD8^+^ T cell infiltration, and boosts the production of the effector cytokine IFN-γ, ultimately alleviating the immunosuppressive state of the TME.

In recent years, combination therapies based on immune checkpoint blockade (ICB) have demonstrated tremendous clinical potential in cancer immunotherapy (42, 43). However, recent studies have revealed that in DLBCL, the positive expression of PD-1 and PD-L1 is predominantly restricted to stromal and immune cells, such as macrophages, within the TME, whereas their expression abundance on the tumor cells themselves is remarkably low (44). Due to this unique immune landscape intertwined with complex evasion mechanisms, the therapeutic benefit of conventional ICB monotherapy in DLBCL has been severely compromised. Consequently, exploring novel combination strategies to remodel the DLBCL immune microenvironment has emerged as an urgent need to break through the current bottlenecks in DLBCL immunotherapy. Herein, we designed and constructed a cRGD peptide-functionalized lipid nanoparticle NOP14-siRNA@LNP. This system leverages the high affinity of cRGD for integrin αvβ3 (28, 29), which is highly expressed on the surface of DLBCL cells, to achieve precise targeted delivery and effective silencing of NOP14. *In vivo* experiments confirmed that NOP14-siRNA@LNP monotherapy significantly suppresses tumor growth. More importantly, it markedly potentiates the synergistic antitumor efficacy of PD-1 blockade by fostering CD8^+^ T cell infiltration and elevating IFN-γ levels within the tumor microenvironment, providing a promising strategy for DLBCL immuno-targeted therapy.

Notwithstanding these encouraging results, several limitations warrant acknowledgment. Firstly, current mouse models do not fully encapsulate the complex heterogeneity of DLBCL, necessitating further validation in complementary models like *Eμ-Myc* transgenic mice. Secondly, although this study demonstrates the regulatory role of NOP14 in the immune microenvironment, the precise molecular mechanisms through which NOP14 modulates the Wnt/β-catenin/c-Myc signaling pathway remain to be fully elucidated. Finally, despite the therapeutic potential of NOP14-siRNA@LNP in xenograft models, its pharmacokinetics and long-term safety profiles require comprehensive assessment prior to clinical translation (45–48). Nevertheless, our findings identify NOP14 as a novel immunomodulatory target, paving the way for new therapeutic strategies in DLBCL.

In summary, our study demonstrates that NOP14 promotes DLBCL proliferation and shapes an immunosuppressive tumor microenvironment by limiting CD8^+^ T cell infiltration and activity. These findings reveal a previously unrecognized mechanism contributing to lymphomagenesis and highlight NOP14 as a clinically viable target to enhance the efficacy of immune checkpoint blockade in DLBCL.

## 5 Conclusion

Collectively, our study illuminated the pivotal role of NOP14 in driving DLBCL progression and established a comprehensive theoretical and experimental foundation for its dual potential as a prognostic biomarker and a novel immunotherapeutic target. In addition, the therapeutic targeting of NOP14 using NOP14-siRNA@LNPs significantly impairs malignant tumor progression while concurrently sensitizing DLBCL cells to immune checkpoint inhibitors, redefining a promising strategic framework for precision immunotherapy.

## Data Availability

Source data for RNA sequencing have been deposited to the NCBI BioProject database, accession: PRJNA1494377.

## Supplementary material

Supplementary Table 1 Primer sequences for RT-qPCR.

Supplementary Table 2 shRNA sequences for NOP14.
