# Supplementary material for: Targeting NOP14 remodels the tumor immune microenvironment and enhances the antitumor efficacy of PD-1 blockade in DLBCL
Source: Front Immunol. 2026 Jul 22;17:1799719. doi: 10.3389/fimmu.2026.1799719 (PMC13438165; doi:10.3389/fimmu.2026.1799719)
Supplement: Supplementary file 1 [file Table1.docx]

Table S1. Primer Sequence for qRT-PCR.

| Gene (Human) | Forward | Reverse |
| --- | --- | --- |
| GAPDH | GGAGCGAGATCCCTCCAAAAT | GGCTGTTGTCATACTTCTCATGG |
| NOP14 | GGACAATCAGTTCCTGGCGA | TGTCCAGTTCCTTGCCTTATT |
