# Supplementary material for: Targeting NOP14 remodels the tumor immune microenvironment and enhances the antitumor efficacy of PD-1 blockade in DLBCL
Source: Front Immunol. 2026 Jul 22;17:1799719. doi: 10.3389/fimmu.2026.1799719 (PMC13438165; doi:10.3389/fimmu.2026.1799719)
Supplement: Supplementary file 2 [file Table2.docx]

**Table S2** **shRNA Sequence for NOP14 and MYC**

| Genes | Species | The Sense Strand | The Antisense Strand |
| --- | --- | --- | --- |
| NOP14 | Human sh#1 | CCTCACAGTCATTGATAAGTT | AACTTATCAATGACTGTGAGG |
|  | Human sh#2 | CAGGAAGCGTACACAGACTTT | AAAGTCTGTGTACGCTTCCTG |
|  | Mouse sh#1 | ACAAACACAAGCGGGAATTTA | TAAATTCCCGCTTGTGTTTGT |
|  | Mouse sh#2 | CTTTACTAAAGGAGTACAAAG | CTTTGTACTCCTTTAGTAAAG |
| MYC | Mouse sh#1 | GAGAATTTCTATCACCAGCAA | TTGCTGGTGATAGAAATTCTC |
