# Supplementary material for: Targeting NOP14 remodels the tumor immune microenvironment and enhances the antitumor efficacy of PD-1 blockade in DLBCL
Source: Front Immunol. 2026 Jul 22;17:1799719. doi: 10.3389/fimmu.2026.1799719 (PMC13438165; doi:10.3389/fimmu.2026.1799719)
Supplement: Supplementary file 3 [file Table3.docx]

**Supplementary Figures**

**Supplementary Figure 1**

**Supplementary Figure 1 Western Blot verification of MYC knockdown and rescue efficiency** (A) Western blot analysis showing the knockdown efficiency of c-Myc (sh#1) relative to the control (shCtrl) in A20 cells. (B) Western blot confirmation of c-Myc re-expression (MYC-OE) in NOP14-knockdown (shNOP14) A20 cells.

**Supplementary Figure 2**

**Supplementary Figure 2 Characterization of NOP14-siCtrl@LNP and NOP14-siRNA@LNP** (A) Particle size distribution and Polydispersity Index (PDI) of NOP14-siCtrl@LNP and NOP14-siRNA@LNP measured by dynamic light scattering. (B) Representative transmission electron microscopy (TEM) images showing the spherical morphology of NOP14-siCtrl@LNP and NOP14-siRNA@LNP. Scale bar:100 nm.

**Supplementary Figure 3**

**Supplementary Figure 3 *In vivo* biosafety evaluation of NOP14-siRNA@LNP in major organs.** Representative H&E staining images of major organs (heart, liver, spleen, lung, and kidney) collected from mice treated with PBS, NOP14-siCtrl@LNP, and NOP14-siRNA@LNP. Scale bars:50 μm.
